# Deep learning-based image reconstruction benefits diffusion tensor imaging for assessing severity of depression

**DOI:** 10.3389/fnins.2025.1607130

**Published:** 2025-08-12

**Authors:** Yuanyuan Cui, Yihao Wang, Weimin Yuan, Youhan Zhang, Yunmeng Wang, Jiankun Dai, Yuxin Cheng, Xin Zhang, Hongbiao Sun, Shuwen Dong, Jinlin Wang, Yonghai Bai, Shiyuan Liu, Yi Xiao

**Affiliations:** ^1^Department of Radiology, Second Affiliated Hospital of Naval Medical University, Shanghai, China; ^2^Department of Psychology, Second Affiliated Hospital of Naval Medical University, Shanghai, China; ^3^Department of Radiology, Qingdao Special Servicemen Recuperation Center of PLA Navy, Qingdao, China; ^4^MR Research, GE Healthcare, Beijing, China

**Keywords:** deep learning, diffusion tensor imaging, depression, white matter tract, fractional anisotropy

## Abstract

**Objective:**

This study aimed to evaluate whether deep learning-based image reconstruction (DLR) improves the accuracy of diffusion tensor imaging (DTI) measurements used to assess the severity of depression.

**Methods:**

A total of 52 patients diagnosed with depression in our hospital between March 2023 and July 2023 were enrolled in this study. The severity of depression was measured using the 9-item Patient Health Questionnaire (PHQ-9). Each patient underwent DTI scans. Two image sets were generated: one with the original DTI (ORI DTI) and one using DLR DTI. Tract-Based Spatial Statistics (TBSS) were used to compare the fractional anisotropy (FA) between DLR DTI and ORI DTI, as well as between patients with mild-to-moderate and those with severe depression. Multivariate logistic regression was carried out to determine independent factors for discriminating mild-to-moderate from severe depression patients. Receiver operating characteristic (ROC) curve analysis and areas under the curve (AUC) were used to assess the diagnostic performance.

**Results:**

Twenty-eight patients with mild-to-moderate depression and 24 with severe depression were included. No significant differences were observed between the two groups in terms of gender (*p* = 0.115), age (*p* = 0.603), or educational background (*p* = 0.148). Compared to patients with mild-to-moderate depression, those with severe depression showed lower FA values in the right corticospinal tract (CST) on ORI DTI. Using DLR DTI, decreases in FA values were observed in the right CST, right anterior thalamic radiation, and left superior longitudinal fasciculus. The diagnostic model based on DLR DTI outperformed the ORI DTI model in assessing severity of depression (AUC: 0.951 vs. 0.764, *p* < 0.001).

**Conclusion:**

DLR DTI demonstrated greater sensitivity in detecting white matter (WM) abnormalities in patients with severe depression and provided better diagnostic performance in evaluating severity of depression.

## HIGHLIGHTS

- Compared to conventional DTI, deep learning-based image reconstruction DTI is more sensitive in detecting WM damage in patients with severe depression.
- The deep learning-based image reconstruction DTI offers more imaging biomarkers than traditional DTI for assessing severity of depression.
- In studies of psychological diseases such as depression, deep learning-based image reconstruction DTI provides a more sensitive approach, particularly when sample sizes are small.

## 1 Introduction

Depression is among the most prevalent psychiatric disorders (Ferrari et al., 2013). According to the World Health Organization (WHO), major depressive disorder (MDD) ranked third in global disease burden in 2018 and is expected to rank first by 2030 (Malhi and Mann, 2018). Assessing the severity of depression at initial diagnosis and during regular follow-up is crucial for guiding clinical management. For patients with moderate depression, defined by a 9-item Patient Health Questionnaire (PHQ-9) score of 10 and 14, antidepressant therapy is typically recommended as a first-line treatment. In case of moderate-to-severe or severe depression (PHQ-9 ≥ 15), a combination of antidepressant medication and psychotherapy is advised (Simon et al., 2024). Currently, the assessment of severity of depression primarily relies on the subjective questionnaire according to the patient's symptoms, with the PHQ being one of the most widely used tools (Levis et al., 2020; Negeri et al., 2021). However, such subjective assessments can be influenced by the clinician's judgment and the patient's understanding (Uher et al., 2008).

Objective and quantitative methods may improve the reliability of assessing severity of depression. White matter (WM) integrity is compromised in patients with depression. It may serve as a biomarker for both the presence (Nothdurfter et al., 2024) and severity (Nobuhara et al., 2006) of the disorder. Diffusion tensor imaging (DTI) is a widely used non-invasive method to evaluate WM integrity (Pilmeyer et al., 2024). However, the conventional diffusion-weighted (DW) sequence, typically performed with single-shot echo-planar imaging (SSEPI), is prone to low signal-to-noise (SNR), which can limit diagnostic accuracy. Multiplexed sensitivity encoding (MUSE) technology was introduced to enable small-field-of-view imaging and multiple excitations, thereby improving signal sampling efficiency and reducing acquisition time and motion artifacts (Chen et al., 2013). MUSE has proved its potential to enhance diagnostic efficacy in other diseases (Wang et al., 2024), though it still exhibits more noise than structural magnetic resonance (MR) sequences. Deep learning-based image reconstruction (DLR) has emerged as a promising approach to enhance MRI quality (Kyathanahally et al., 2018; Loizillon et al., 2024), with several manufacturers now offering DLR for clinical use. Studies show that applying DLR to MUSE diffusion-weighted imaging (DWI) can yield more robust phase estimation and higher resolution (Zhang et al., 2021). Because of its high SNR, DLR may enhance the accuracy of DTI-based WM assessments. Nevertheless, its role in evaluating the severity of depression remains unexplored.

This study investigated the value of DLR DTI for measuring WM damage in patients with depression and explored its potential to differentiate the severity of depression. These findings may support the use of DLR DTI as a quantitative tool for assessing the severity of psychiatric diseases such as depression.

## 2 Methods and materials

### 2.1 Population and psychological assessment

This paper retrospectively enrolled patients with clinically confirmed depression who underwent DTI scans at our hospital from April to July 2023. Face-to-face assessments of depression, anxiety, and insomnia were conducted, followed by the MRI within 1 week.

Inclusion criteria were as follows: (1) a clinical diagnosis with a PHQ-9 score above 4; (2) right-handed patients; (3) age over 18 years. Exclusion criteria were as follows: (1) brain abnormalities (tumors, hemorrhages, infarcts) detected by conventional MRI; (2) a current or past history of severe physical or neurological diseases; (3) a family history of severe mental or neurological diseases in first-degree relatives; (4) a current or past history of psychiatric conditions such as depression, anxiety, or substance abuse; (5) contraindications to MRI; (6) DTIs with noticeable artifacts that could affect the investigation of the images. Age, gender, and educational background were recorded, with education categorized into four groups: below senior high school, senior high school, college graduate, or above college graduate. A total of 52 patients (mean age 51.020 ± 12.379 years) were included in this study, with 18 males and 34 females.

Psychological assessments were carried out under the supervision of a psychiatrist with over 5 years of experience to ensure reliability and consistency. Severity of depression was evaluated using the PHQ-9 based on the Diagnostic and Statistical Manual of Mental Disorders, Fifth Edition (DSM-5), with patients categorized into mild to moderate depression or severe depression groups using a cutoff value of 15 (Kroenke et al., 2001). Anxiety symptoms were assessed with the Generalized Anxiety Disorder-7 (GAD-7) scale (Spitzer et al., 2006), and insomnia was measured using the Athens Insomnia Scale (AIS) (Soldatos et al., 2000). This study was approved by the ethics committee of the Second Affiliated Hospital of Naval Medical University, and all patients provided informed consent.

### 2.2 MRI acquisition

MR images were acquired using a 3.0 T MRI scanner (Signa Premier; GE Healthcare, Milwaukee, WI, USA) with a 48-channel phased-array head coil. Each participant received T2WI and DTI. To alleviate distortion and susceptibility artifacts, DTI was imaged using the advanced MUSE DWI technique. T2WI was scanned with repetition time (TR) = 6,505 ms, echo time (TE) = 103.4 ms, field of view (FOV) = 24 × 24 cm^2^, matrix size = 512 × 512, slice thickness = 5.0 mm, slice gap = 0 mm, number of slices = 30, and number of excitation (NEX) = 1. DTI was acquired with TR = 5,500 ms, TE = 57.5 ms, FOV = 24 × 24 cm^2^, matrix = 160 × 160, slice thickness = 3.0 mm, number of slices = 50, *b*-value = 0 and 1,000 s/mm^2^, number of diffusions weighting directions = 30, and NEX = 2. The DW images of DTI were separately reconstructed using the original (i.e., conventional inverse Fourier transformation) and a vendor-provided, commercially available deep learning algorithm (AIR™ Recon DL; GE Healthcare, USA). The AIR™ Recon DL pipeline included a deep convolutional neural network (CNN) that operated on raw, complex-valued imaging data to produce a clean output image. The CNN contained 4.4 million trainable parameters in ~10,000 kernels and was trained using a supervised learning approach with a training database of 4 million unique image/augmentation combinations. Training was performed in a single epoch of the training database. The ADAM optimizer was used to minimize the loss between the predicted and near-perfect images. A generative adversarial network was not used to enhance image sharpness, thereby avoiding potential hallucinations of new features (Lebel, 2020).

### 2.3 Image analysis

#### 2.3.1 Image signal evaluation

In the first DWI images with a *b*-value of 1,000 from DLR DTIs, regions of interest (ROIs) were delineated in the right frontal cortex and the WM of the semioval center level. An additional ROI was delineated in the right frontal background at the same level. These ROIs were then copied onto the ORI DTI to maintain consistent size and location. The delineation process was illustrated in Supplementary Figure 1. The ROI delineation was performed by two radiologists with more than 5 years of diagnostic experience. Discrepancies were resolved through discussion to reach consensus. The average signal value and standard deviation (SD) with each ROI were recorded as measures of tissue signal and noise. The SNR and contrast-to-noise ratio (CNR) for the WM and cortex were calculated for both DW images of ORI and DLR DTI using the following formulas:


$$\begin{array}{l} \text{SNR }=\text{ S}{\text{I}}_{\text{brain tissue}}/\text{S}{\text{D}}_{\text{background}}\\ \text{CNR }=\left(\text{S}{\text{I}}_{\text{brain tissue}}-\text{ S}{\text{I}}_{\text{background}}\right)/\\ \sqrt{{\text{S}{\text{D}}_{\text{brain tissue}}}^{2}+{\text{S}{\text{D}}_{\text{background}}}^{2}} \end{array}$$

#### 2.3.2 Quantitative analysis of DTI

Raw Digital Imaging and Communications in Medicine (DICOM) images were converted to Neuroimaging Informatics Technology Initiative (NIfTI) using dcm2niix (https://github.com/rordenlab/dcm2niix). The image data were analyzed using Tract-Based Spatial Statistics (TBSS) and the FMRIB software library (FSL, version 4.1.8; http://www.fmrib.ox.ac.uk/fsl). The fractional anisotropy (FA) image was calculated through the following steps. First, susceptibility distortions were corrected with the top-up tool. Second, motion and eddy current distortions were corrected using the eddy correct tool. Third, brain masks were extracted from the b0 image with the FSL brain extraction tool. Fourth, the FA was produced with the DTIFIT. Each patient's FA image was registered to the FMRIB58_FA template in the Montreal Neurological Institute (MNI) space using FNIRT, FMRIB's non-linear registration tool. A mean FA image was calculated from all aligned maps, and a mean FA skeleton was generated using a threshold value of 0.2. Finally, each patient's FA map was projected onto the skeleton.

Voxel-wise differences in the skeletonized FA between the DLR and ORI DTI were compared using paired two-sample *t*-tests. These differences between patients with mild-to-moderate and severe depression were assessed using two-sample *t*-tests. The analysis was performed with 5,000 permutations and the threshold-free cluster enhancement (TFCE) in FSL's randomize tool (version 2.1), incorporating age and gender as covariates to minimize their potential effects. Statistical significance was defined as *p* < 0.05 (two-sided, family-wise error [FWE] corrected). The WM regions showing significant differences in the DTI metrics were overlaid onto the Johns Hopkins University (JHU) WM Tractography Atlas. ROIs were then defined *post-hoc* based on statistically significant clusters (FWE-corrected *p* < 0.05), which were converted into binary masks. Due to the alignment of significant cluster masks, individual skeletonized FA data, and statistical maps within the same MNI space (from initial FNIRT registration and TBSS projection), the masks were directly applied to each subject's skeletonized FA image without further spatial transformation. Mean FA values within each ROI were extracted for each subject using fslmeants for subsequent *post-hoc* analysis.

### 2.4 Statistical analysis

Unpaired two-sample *t*-tests were performed to evaluate age and psychological test scores between patients with mild-to-moderate and severe depression. Kruskal–Wallis tests were employed for non-normally distributed samples. A chi-square test was utilized to assess differences in sex distribution, while an appropriate non-parametric test was applied to compare educational background. Signal, noise, SNR, and CNR of WM and gray matter (GM) were compared between the ORI and DLR DTI. Paired *t*-tests were used to assess differences in FA across the whole brain between ORI and DLR DTI. Mean diffusion metrics were then extracted from brain regions that showed significant differences between the two image types.

Two-sample *t*-tests were used to compare FA values between two groups of patients. Pearson correlation analysis was applied for normally distributed data, whereas Spearman correlation was employed for non-normally distributed data to assess the relationships between diffusion parameters and psychological test scores. Correlation coefficients were interpreted as mild (0.2–0.4), moderate (0.4–0.7), or strong (0.7). Univariate logistic regression was performed to calculate odds ratios (ORs) and 95% confidence intervals (CIs) for FA values in various differentiated brain regions, assessing their diagnostic performance in distinguishing severity of depression. Additionally, the univariate logistic regression models were constructed based on the FAs of different brain regions. A multivariate logistic regression model was conducted using FAs from different brain regions derived from DLR DTI to assess severity of depression. Model performance was assessed using the receiver operating characteristic (ROC) curve by calculating the areas under the curve (AUCs). The Delong test was used to evaluate the differences among models. Statistical power was calculated at a significance level of 0.05 using R software provided functions “pwr.t2n.test,” “power.roc.test,” and “pwr.f2.test” for parameter comparisons, ROC analysis, and multivariate regression, respectively. The predictive performance of the univariable logistic regression models was further assessed using leave-one-out cross-validation (LOOCV) implemented in R software with the “caret” package. In this approach, each model was iteratively trained on n-1 samples and validated on the excluded sample. All analyses were performed using SPSS 26.0 (IBM Corp., Armonk, NY) and R software 2024.04.2 (Posit Software, PBC), with *p* < 0.05 considered statistically significant.

## 3 Results

### 3.1 Patient characteristics

Among all patients, there were 28 cases of mild-to-moderate depression and 24 cases of severe depression. No significant differences were observed between the two groups in terms of gender (male/female: 7/21 vs. 11/13, *p* = 0.115), age (51.857 ± 14.656 vs. 50.042 ± 9.262, *p* = 0.603), or education level (*p* = 0.148). However, significant differences were found in depression scores (17.583 ± 2.145 vs. 8.643 ± 2.599, *p* < 0.001), anxiety scores (16.167 ± 2.959 vs. 7.643 ± 2.376, *p* < 0.001), and insomnia scores (16.250 ± 3.082 vs. 10.179 ± 2.763, *p* < 0.001), as summarized in Table 1.

**Table 1:** The demographic characteristics of the patients.

**Demographic characteristics**	**Mild to moderate depression (mean ±SD/*n*) (*n* = 28)**	**Severe depression (mean ±SD/*n*) (*n* = 24)**	***p*-value**
Age (year)	50.042 ± 9.262	51.857 ± 14.656	0.603
Gender (female/male)	21/7	11/13	0.115
Educational background (below senior high school/senior high school/graduate/above graduate)	1/19/6/1	1/18/5/0	0.148
PHQ-9	8.643 ± 2.599	17.583 ± 2.145	< 0.001
GAD-7	7.643 ± 2.376	16.167 ± 2.959	< 0.001
AIS	10.179 ± 2.763	16.250 ± 3.082	< 0.001

PHQ, Patient Health Questionnaire; GAD, Generalized Anxiety Disorder; AIS, Athens Insomnia Scale; SD, standard deviation.

### 3.2 DLR and ORI DTI signals

**Figure 1:**
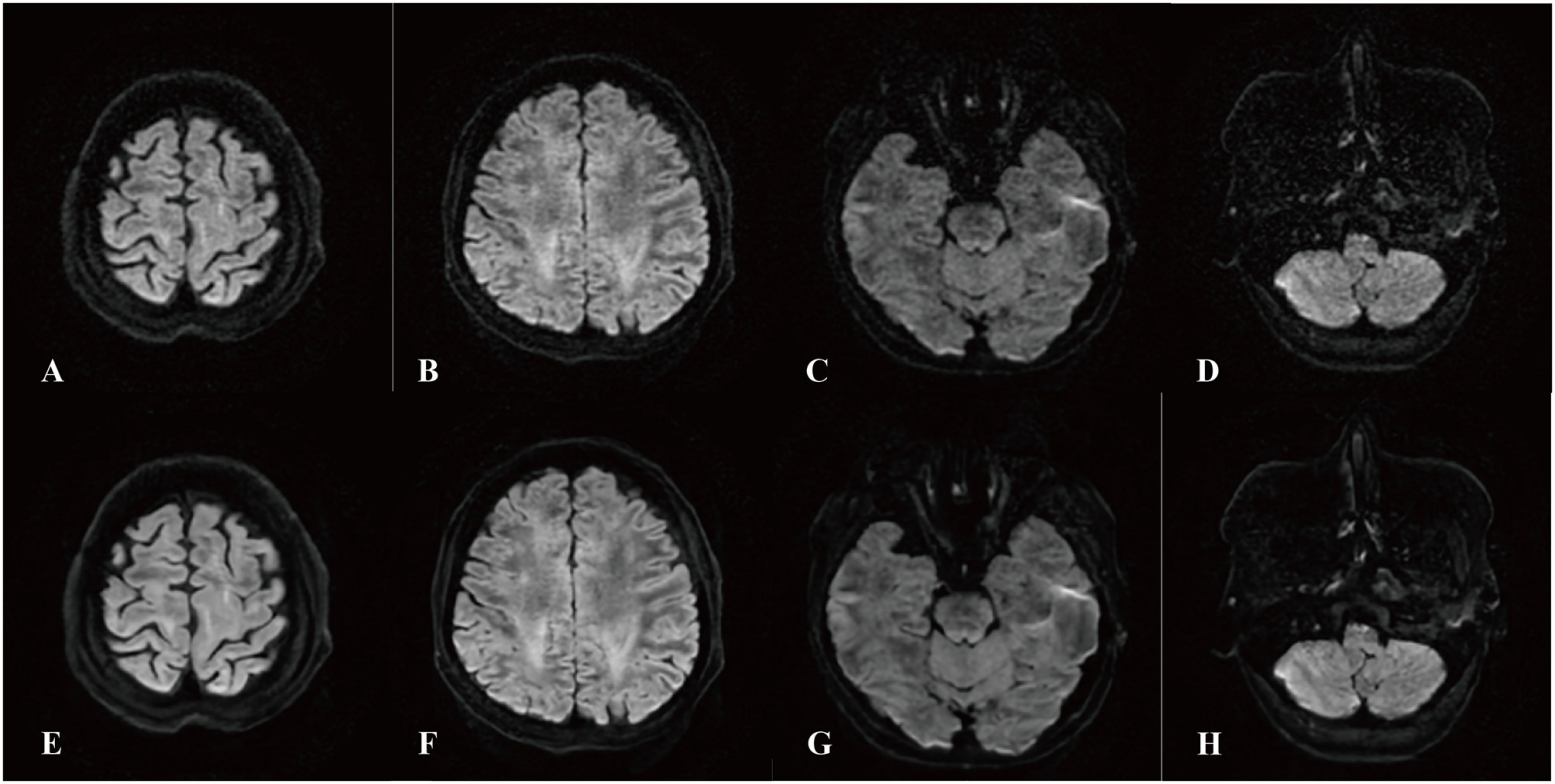
Comparison of deep learning post-reconstruction diffusion tensor images (DLR DTI) and original DTI (ORI DTI). **(A–D)** Show ORI DTI, and **(E–H)** show DLR DTI. The images were derived from the same patient and demonstrated significant noise reduction in ORI DTI after DL post-reconstruction. In both sequences, visual inspection reveals a reduction of noise in the parenchyma, extracranial tissues, and background. The interface of gray and white matter is clearer in DLR DTI, even at the skull base, where bone structure and air space always affect the magnetic field.

The signal intensities in WM and GM regions on DLR DTI were slightly lower than those on ORI DTI, but these differences were not statistically significant (1030.538 ± 175.730 vs. 1037.494 ± 184.817, *p* = 0.110; 1092.458 ± 178.225 vs. 1099.863 ± 185.227, *p* = 0.080). In contrast, noise levels in both WM and GM were significantly lower on DLR DTI compared to ORI DTI (77.978 ± 30.912 vs. 94.034 ± 32.281, *p* < 0.001; 50.904 ± 32.349 vs. 66.747 ± 26.691, *p* < 0.001). The background signal was also significantly reduced on DLR DTI (33.929 ± 12.813 vs. 43.622 ± 13.421, *p* < 0.001). Compared to ORI DTI, the DLR DTI showed higher SNR (66.068 ± 71.134 vs. 43.324 ± 51.976, *p* < 0.001; 69.208 ± 70.139 vs. 45.579 ± 50.882, *p* < 0.001) and CNR (14.676 ± 8.665 vs. 10.629 ± 4.064, *p* < 0.001; 22.764 ± 10.363 vs. 15.772 ± 5.046, *p* < 0.001) in WM and GM. Figure 1 illustrates the ORI and DLR DTI images, highlighting the DLR algorithm's ability to reduce noise across all regions, including the parenchyma, extracranial tissues, and background, thereby substantially improving the overall image quality of DTI.

### 3.3 Differences of FA between DLR and ORI DTI

**Figure 2:**
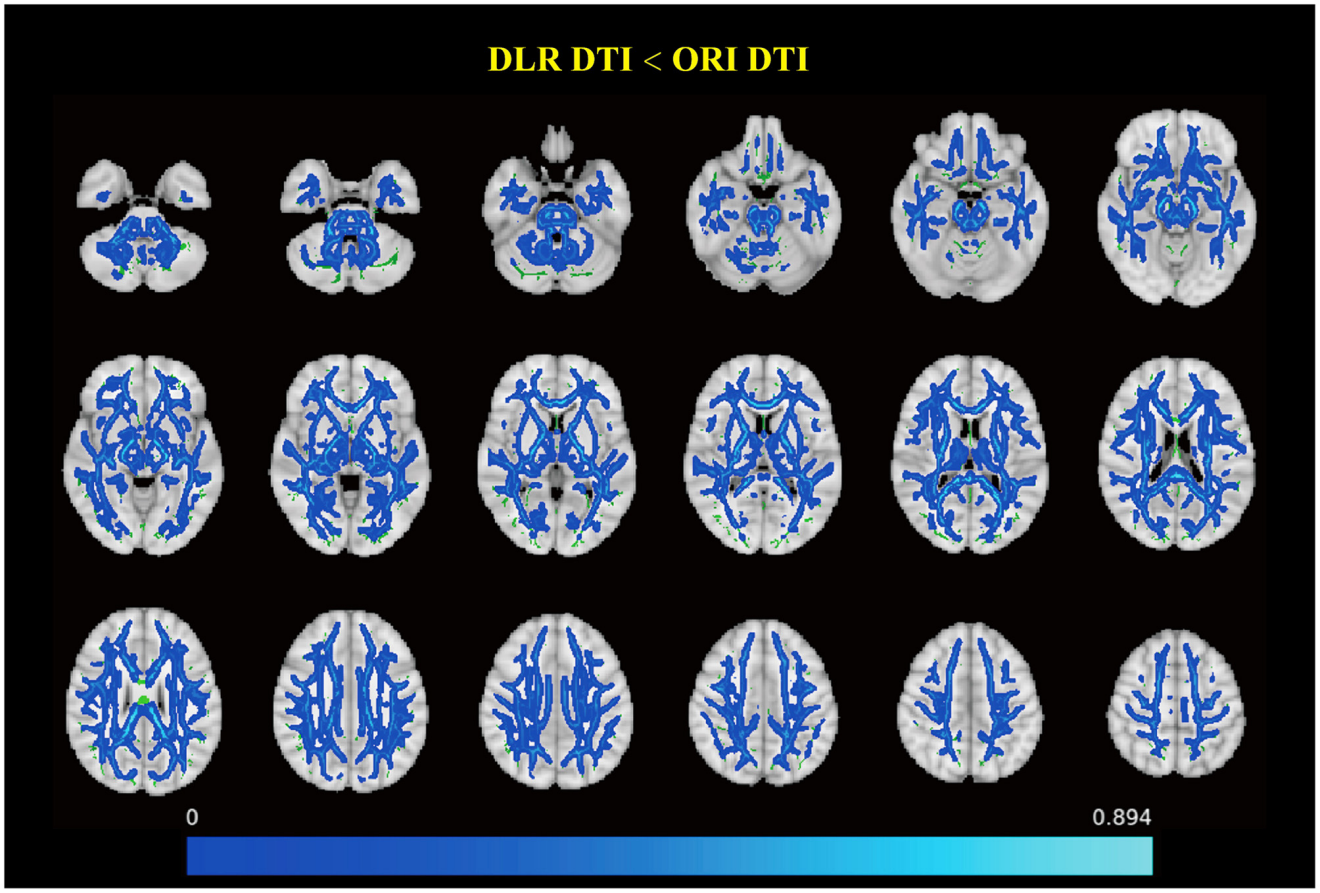
Comparison of fractional anisotropy (FA) between deep learning post-reconstruction diffusion tensor images (DLR DTI) and original DTI (ORI DTI). It represents the different regions of FA between DLR DTI and ORI DTI. The blue regions show that the FAs of DLR DTI are smaller than those of ORI DTI in multiple brain regions. DLR DTI, deep learning post-reconstruction diffusion tensor images; ORI DTI, original diffusion tensor images.

The brain regions with significant differences are shown in Figure 2 and Supplementary Tables 1, 2. Among these, six clusters exceeded 50 voxels, with the largest comprising 86,785 voxels. The mean FA of this largest cluster was significantly lower when measured using DLR DTI compared to ORI DTI (0.488 ± 0.014 vs. 0.500 ± 0.014, *p* < 0.001). Overall, FA values obtained from DLR DTI were reduced across most WM regions relative to those from ORI DTI.

### 3.4 Differentiating mild-to-moderate and severe depression based on DLR and ORI DTI

**Figure 3:**
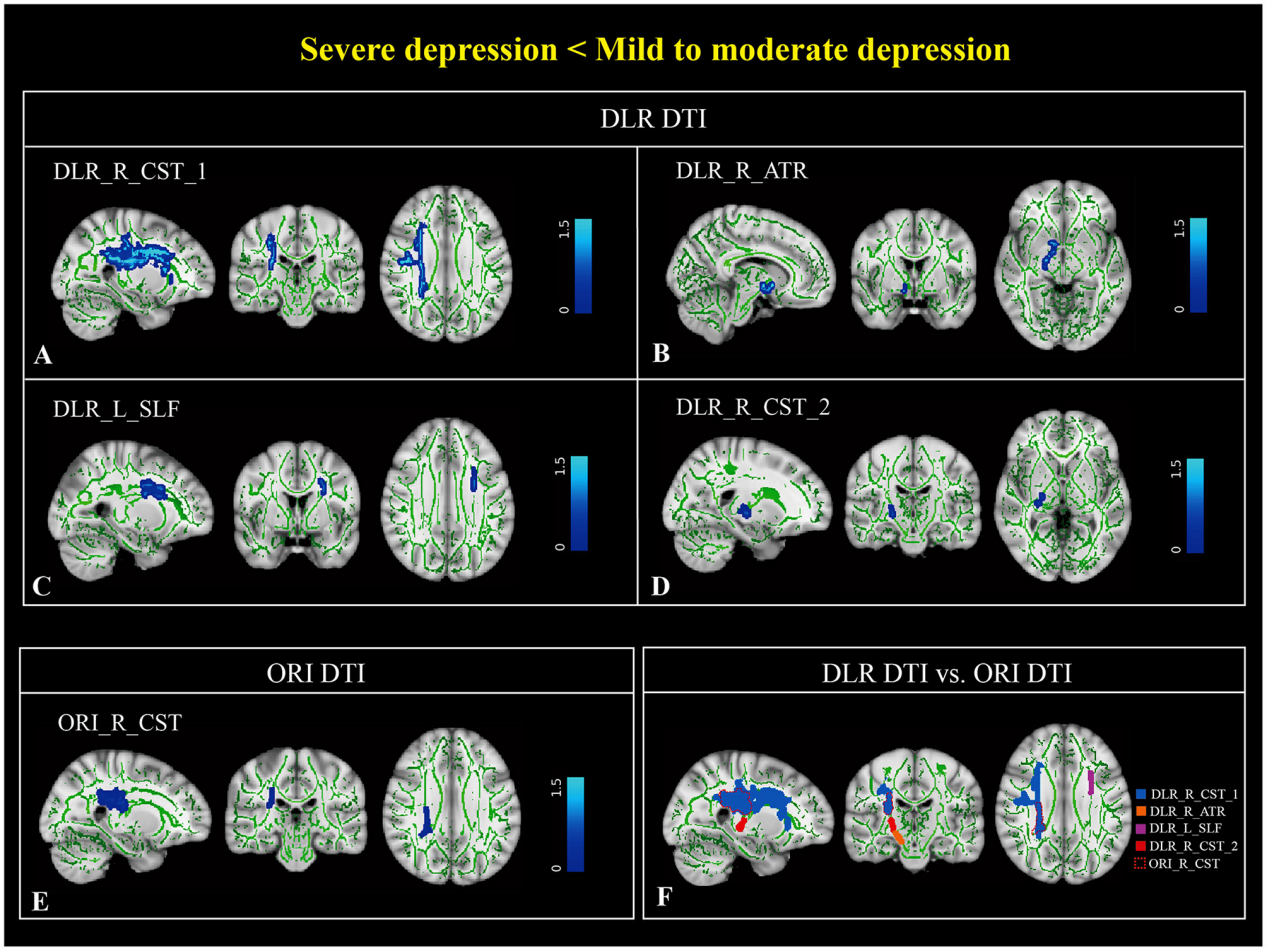
Differential brain regions of fractional anisotropy (FA) derived from deep learning post-reconstruction diffusion tensor images (DLR DTI) and original DTI (ORI DTI) in patients with severe and those with mild-to-moderate depression. Based on DLR DTI analysis, four differential brain regions were found between the two groups, located in the right corticospinal tract_1 (DLR_R_CST_1), right anterior thalamic radiation (DLR_R_ATR), left superior longitudinal fasciculus (DLR_L_SLF), and right corticospinal tract_2 (DLR_R_CST_2) **(A–D)**. Based on ORI DTI analysis, only one differential brain region was found between the two groups, located in the right corticospinal tract (ORI_R_CST) **(E)**. In **(F)**, blue, orange, purple, and red represent the DLR_R_CST_1, DLR_R_ATR, DLR_L_SLF, and DLR_R_CST_2, respectively. In the fusing images, it can be observed that most parts of the ORI_R_CST (dashed line) overlapped with DLR_R_CST_1 (blue). DLR DTI, deep learning post-reconstruction diffusion tensor images; ORI DTI, original diffusion tensor images; DLR_R_CST_1, deep learning post-reconstruction_right corticospinal tract_1; DLR_R_ATR, deep learning post-reconstruction_right anterior thalamic radiation; DLR_L_SLF, deep learning post-reconstruction_left superior longitudinal fasciculus; ORI_R_CST, original_right corticospinal tract.

**Figure 4:**
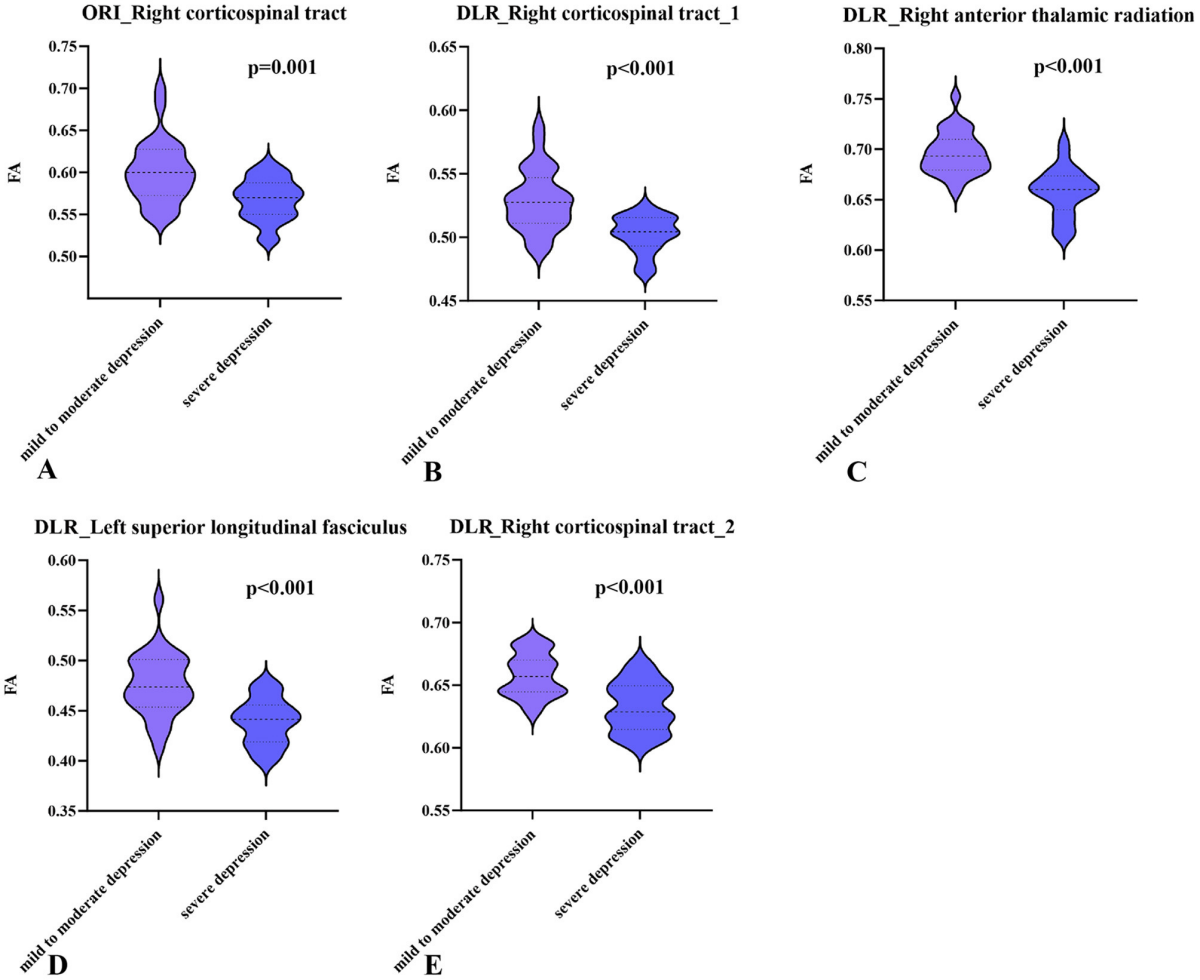
Comparison of the extracted mean fractional anisotropy (FA) values from the differential white matter between the patients with severe and those with mild-to-moderate depression: **(A–D)** show the comparisons of the extracted mean FA values of different white matter regions based on the deep learning post-reconstruction diffusion tensor images **(**DLR DTI) between the two groups; **(E)** shows the comparisons of the extracted mean FA values of different white matter regions based on the original DTI (ORI DTI) between the two groups. FA, fractional anisotropy; ORI, original; DLR, deep learning post-reconstruction.

Based on DLR DTI, differences in FA between patients with mild-to-moderate and severe depression were observed in the right corticospinal tract (CST), left superior longitudinal fasciculus (SLF), and right anterior thalamic radiation (ATR) (Table 2, Figures 3A–D). The differential brain regions identified based on ORI DTI were in the right CST (Figure 3E). The different regions analyzed based on ORI DTI were included within the regions analyzed based on DLR DTI (Figure 3F). A comparison of FA extracted from the differential brain regions is shown in Figure 4.

**Table 2:** The significantly different regions of white matter in fractional anisotropy (FA) between the two groups (mild to moderate depression > severe depression).

**Cluster**		**Side**	**Brain regions (JHU)**	**Voxel size**	**MNI coordinate (max vox)**			***p*-value**
					**X (mm)**	**Y (mm)**	**Z (mm)**	
ORI DTI	1	R	Corticospinal tract	698	34	−48	12	0.041
DLR DTI	1	R	Corticospinal tract_1	2,943	34	−35	12	0.023
DLR DTI	2	R	Anterior thalamic radiation	275	11	7	−5	0.048
DLR DTI	3	L	Superior longitudinal fasciculus	274	−26	13	29	0.040
DLR DTI	4	R	Corticospinal tract_2	92	27	−23	1	0.049

ORI DTI, original diffusion tensor imaging; DLR DTI, deep learning-based image reconstruction diffusion tensor imaging; L, left; R, right; Corticospinal tract_1, corticospinal tract cluster 1; Corticospinal tract_2, corticospinal tract cluster 2; OR, odd ratio; CI, confidence interval; JHU, Johns Hopkins University White-Matter Tractography Atlas; MNI, Montreal Neurological Institute.

**Figure 5:**
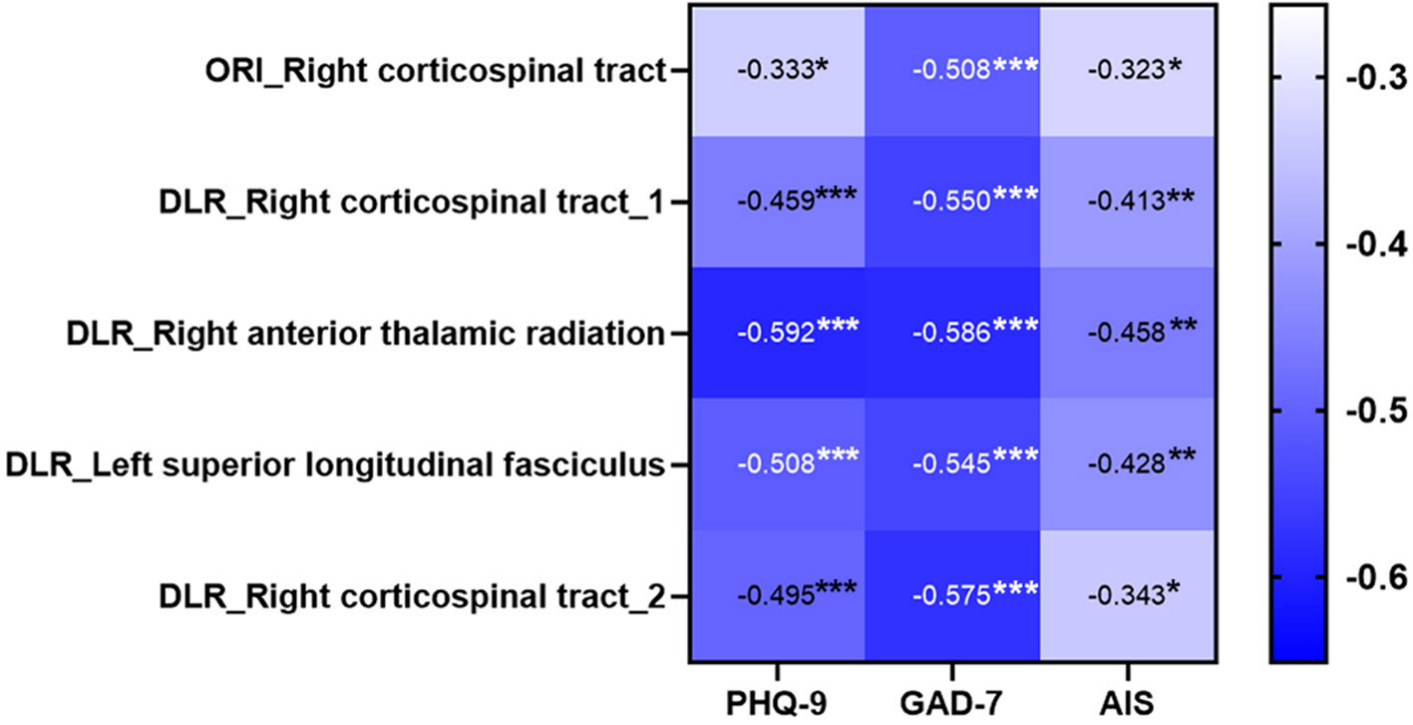
The heatmap of the correlation between each differential brain region and depression, anxiety, and sleep. The fractional anisotropy (FA) of the right anterior thalamic radiation based on deep learning post-reconstruction diffusion tensor images (DLR DTI) had the highest correlation with depression scores. *Means *p* < 0.05, **means *p* < 0.01, ***means *p* < 0.001. ORI, original; DLR, deep learning post-reconstruction.

The FA in all brain regions was negatively correlated with scores on PLC-9, GAD-7, and AIS (Figure 5). In terms of correlation with PLC-9, the FA of the right ATR based on DLR DTI had a moderate and the highest correlation (*r*^2^ = −0.592, *p* < 0.001), whereas the FA of the right CST based on ORI DTI had a mild and the lowest correlation (*r*^2^ = −0.333, *p* = 0.016).

Univariate logistic regression was performed to evaluate the diagnostic efficacy of each parameter in differentiating mild-to-moderate and severe depression (Table 3). Among these, the FA of the right ATR based on DLR DTI showed the highest odds ratio (OR = 0.678, 95% CI: 0.517, 0.796), while the FA of the right CST based on ORI DTI had the lowest (OR = 0.505, 95% CI: 0.416, 0.587). The univariate logistic regression models were built to differentiate between mild-to-moderate and severe depression (Table 4). The model based on the FA of the right CST from ORI DTI achieved an AUC of 0.764 (95% CI: 0.637, 0.891). According to the DeLong test, the model utilizing the FA of the right ATR from DLR DTI demonstrated significantly better performance than the ORI DTI-based CST model (*p* = 0.042). However, models incorporating the FAs of the right CST cluster 1, left SLF, or right CST cluster 2 based on DLR DTI showed no differences with the ORI DTI-based CST model (*p* = 0.110, *p* = 0.362, and *p* = 0.408).

**Table 3:** The contribution of DLR DTI and ORI DTI in distinguishing mild to moderate depression from severe depression.

**Factors**		**Univariate logistic regression**		**Multivariate logistic regression**	
		**OR (95% CI)**	***p*** **-value**	**OR (95% CI)**	***p*** **-value**
ORI DTI	ORI_Right corticospinal tract	0.505 (0.416, 0.587)	0.001	–	–
DLR DTI	DLR_Right corticospinal tract_1	0.644 (0.485, 0.765)	0.001	3.020 (0.586, 9.828)	0.787
DLR DTI	DLR_Right anterior thalamic radiation	0.678 (0.517, 0.796)	< 0.001	0.599 (0.394, 0.754)	0.028
DLR DTI	DLR_Left superior longitudinal fasciculus	0.556 (0.452, 0.649)	0.001	0.566 (0.374, 0.718)	0.043
DLR DTI	DLR_Right corticospinal tract_2	0.617 (0.482, 0.727)	0.001	2.140 (0.690, 4.771)	0.457

ORI DTI, original diffusion tensor imaging; DLR DTI, deep learning-based image reconstruction diffusion tensor imaging; corticospinal tract_1, corticospinal tract cluster 1; corticospinal tract_2, corticospinal tract cluster 2; OR, odd ratio; CI, confidence interval.

**Table 4:** The performance of the model based on DLR DTI and ORI DTI in distinguishing mild to moderate depression from severe depression.

**Model**		**AUC (95%CI)**	**ACC**	**SE**	**SP**
ORI DTI	ORI_Right corticospinal tract model	0.764 (0.637, 0.891)	0.692	0.750	0.643
DLR DTI	DLR_Right corticospinal tract_1 model	0.832 (0.721, 0.943)	0.788	0.958	0.643
DLR DTI	DLR_Right anterior thalamic radiation model	0.900 (0.813, 0.987)	0.846	0.833	0.857
DLR DTI	DLR_Left superior longitudinal fasciculus model	0.830 (0.720, 0.941)	0.788	0.708	0.857
DLR DTI	DLR_Right corticospinal tract_2 model	0.814 (0.699, 0.929)	0.769	0.667	0.857
DLR DTI	DLR_Combined model	0.951 (0.898, 1.000)	0.856	0.776	0.958

The DLR combined model was built using values derived from all the different brain regions, based on deep learning post-processing diffusion tensor images and adjusted for age, gender, and educational background. ORI DTI, original diffusion tensor imaging; DLR DTI, deep learning-based image reconstruction diffusion tensor imaging; corticospinal tract_1, corticospinal tract cluster 1; corticospinal tract_2, corticospinal tract cluster 2; CI, confidence interval; AUC, area under curve; ACC, accuracy; SE, sensitivity; SP, specificity.

**Figure 6:**
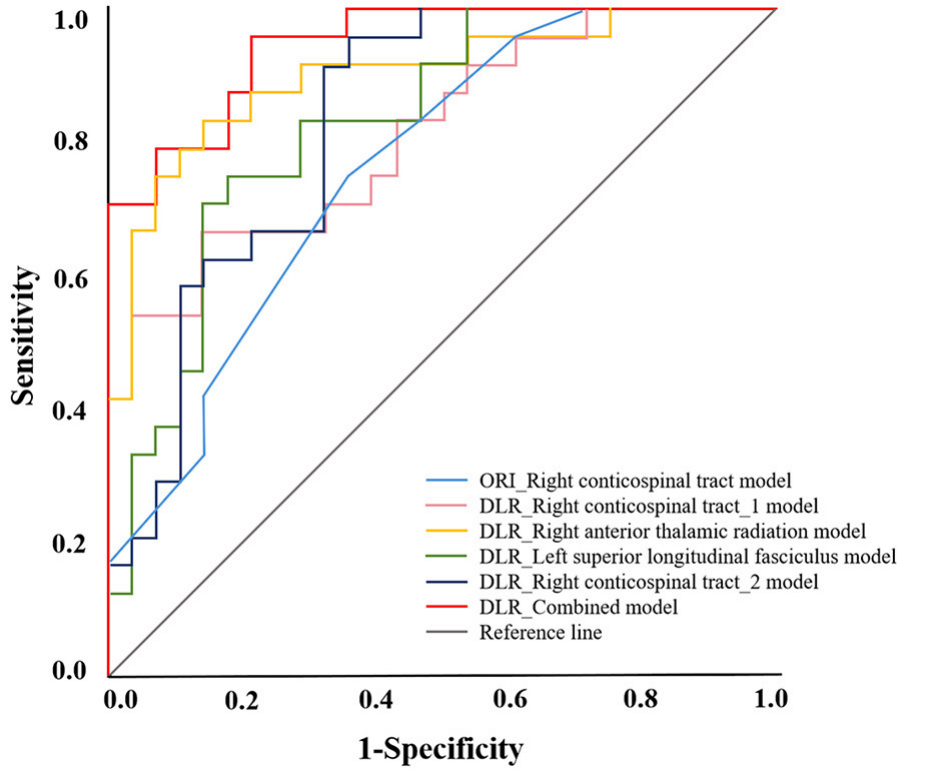
The Receiver Operating Characteristic (ROC) curve of the severity of depression assessment model is based on fractional anisotropy (FA) values in different brain regions. The DLR combined model, which incorporates FA values from all differential brain regions based on deep learning post-reconstruction diffusion tensor images (DLR DTI) (indicated by the red line), has the largest area under the curve. ORI, original; DLR, deep learning post-reconstruction.

Combining all the FAs of differential brain regions identified by DLR DTI into a single model resulted in an AUC of 0.951 (95% CI 0.898, 1.000) (Table 4, Figure 6). The right ATR and left SLF were included in this combined model. LOOCV demonstrated that the DLR-combined model achieved an AUC of 0.885. Comprehensive performance metrics for all models, validated by LOOCV, are presented in Supplementary Table 3. The Delong test showed that the combined model outperformed the model based on ORI DTI (*p* = 0.001).

The statistical power exceeded 0.90 for all parameter comparisons between patients with mild-to-moderate and severe depression, except for ORI DTI, which showed a power of 0.87. Similarly, the statistical power of all ROC analyses involving single parameters was above 0.90, whereas the multivariate regression analysis had a power of 0.81. These findings indicated that all logistic regression models demonstrated good statistical power.

## 4 Discussion

This study demonstrated the potential of DLR DTI for assessing depression. DLR significantly enhanced the signal-to-noise ratio (SNR) and contrast-to-noise ratio (CNR) of DTI images. By reducing noise, DLR DTI was more effective than ORI DTI in detecting damaged WM tracts in patients with severe depression. Furthermore, a combined model using DLR DTI outperformed the model based on ORI DTI in distinguishing between mild to moderate and severe depression.

Following DLR, a significant noise reduction was observed in both WM and GM on DTI images in the present study. Specifically, the SNR in WM and GM increased by about 50%, whereas the CNR improved by ~40% compared to the original images. The noise reduction significantly enhanced tissue resolution. As shown in Figure 2, DLR DTI improved the delineation between GM and WM, resulting in sharper boundaries and facilitating easier tissue differentiation. This improvement also enabled more precise delineation of ROIs, which could benefit the image post-processing of the DTI.

As Denis Le Bihan et al. noted, SNR could impact the quantification of DWI (Iima et al., 2020). Both simulation-based and *in vivo* studies have examined the impact of noise on FA indices in DTI (Pierpaoli and Basser, 1996; Bastin et al., 1998; Anderson, 2001; Jones and Basser, 2004; Landman et al., 2007). These studies demonstrated that low SNR leads to an upward bias in measured FA values. This bias contributed to the overestimation of axial diffusivity (AD) and the underestimation of radial diffusivity (RD) (Pierpaoli and Basser, 1996; Bastin et al., 1998; Anderson, 2001; Jones and Basser, 2004; Landman et al., 2007). The DLR used in this study significantly improved the SNR of DTI images. Consistently, we observed that higher SNR after DLR was associated with lower FA indices of DTI, accompanied by decreased AD and increased RD (Supplementary Figure 2). Similarly, a recent study reported considerably lower FA value in the femur and tibia growth plates after applying the same DLR algorithm (Santos et al., 2024). Other studies have employed different deep learning algorithms to denoise DW images and reported notably reduced FA values of DTI (Sagawa et al., 2021; Pouliquen et al., 2024). The CNR of DLR DTI was also improved. The DLR algorithm primarily optimizes images by reducing background noise and significantly mitigating Gibbs artifacts caused by K-space undersampling. After DLR processing, background noise around lesions was significantly reduced without compromising tissue signals, thereby improving the image quality. The enhanced tissue contrast improves quantitative diagnosis and disease differentiation. Consequently, quantitative analyses based on DLR DTI may provide more accurate tissue characterization across various pathological conditions, ultimately supporting more reliable disease evaluation.

In this study, analysis of FA derived from DLR DTI identified four distinct brain regions that differentiated patients with mild-to-moderate depression from those with severe depression. These regions included the right CST, right ATR, and left SLF. Among them, the right ATR demonstrated the strongest correlation with severity of depression. Previous research has extensively investigated WM tract damage in depression. For example, Ahn et al. reported damage to multiple WM tracts, including the forceps major, left CST, left SLF-parietal bundle, right ATR, and right SLF-temporal bundle, in MDD patients compared to healthy controls (Ahn et al., 2024). Several studies with limited sample sizes have also indicated WM integrity impairment in patients with MDD (Bracht et al., 2015). However, due to potential limitations in generalization and reliability, larger sample sizes have become standard in this field. For instance, Velzen et al. analyzed a large DTI dataset comprising 1,305 depression patients and 1,602 healthy controls to identify and rank the most robustly impaired WM (van Velzen et al., 2020). Their results indicated that the corona radiata, corpus callosum, and internal capsule (a part of the CST) were among the most affected regions. Similarly, a large-sample study by Shen et al., utilizing data from the United Kingdom Biobank Imaging Study, found that reduced WM microstructure in the ATR was significantly associated with multiple depressive symptoms. Specifically, the severity of depression was linked to decreased WM integrity in the association fibers and thalamic radiations (Shen et al., 2019). These findings strongly implicate the ATR in the development of depression. The ATR is a component of the limbic-thalamo-cortical circuit (Li et al., 2023) and has been linked to reward-seeking and punishment-related functions in the human brain (Coenen et al., 2012). Additionally, the integrity of ATR may influence the development of MDD through brain circuitry involved in cognitive control (Sanjuan et al., 2013). Moreover, a study investigating Brain-Derived Neurotrophic Factor (BDNF) reported a correlation between this gene and ATR damage in patients with severe depression (Choi et al., 2015). BDNF plays a pivotal role in neuronal plasticity, differentiation, survival, and function (Dwivedi, 2009). The discovery further underscores the role of ATR disruption in severe depression.

Previous studies have demonstrated ATR damage in patients with depression. However, in our small-sample study, analysis based on ORI DTI failed to identify these crucial brain regions. When we applied DLR DTI, which improved tissue SNR, to the same sample size, it demonstrated greater sensitivity in detecting ATR as a vital region associated with severe depression. Additionally, our DLR DTI-based analysis revealed damage to the left SLF in patients with severe depression, showing a negative correlation between severity of depression and left SLF integrity. This result aligns with previous studies (Cole et al., 2012; Guo et al., 2012; Lai and Wu, 2014). Similar to the ATR, this WM region was not detected using ORI DTI, potentially due to noise interfering with the accuracy of FA calculations compared to DLR DTI. For the brain region identified differently by both methods, the right CST and its voxels identified by ORI DTI were encompassed within those detected by DLR DTI. However, the correlation between extracted values and severity of depression was lower, underlining DLR DTI's superior sensitivity in identifying differential brain regions. Furthermore, the severity of depression assessment model constructed using differential brain regions identified by DLR DTI demonstrated substantially higher predictive efficiency than the ORI DTI-based model, offering robust performance and significant clinical value for accurately evaluating patient conditions.

This study has several limitations. First, although the statistical power with the sample used in this study was acceptable, the preliminary results based on a small sample size may limit the generality. Larger studies are required to validate these findings. Second, while the advanced MUSE reduces distortions and susceptibility artifacts of DWI compared to conventional single-shot EPI DTI, it doubles the acquisition time under identical imaging parameters. Previous studies have reported that the DLR used in this study can accelerate the acquisition of liver DWI without compromising diagnostic performance compared with standard slow acquisition (Zhu et al., 2025). Therefore, further study should investigate the feasibility of using DLR to reduce MUSE DTI acquisition times and evaluate its impact on assessing the severity of depression. Thirdly, due to the limited sample size, this preliminary study did not compare multiple machine learning models (such as support vector machines, random forests, or gradient boosting) to identify the most effective method for leveraging DTI data. This will be addressed in future studies as more samples are collected. Finally, the diagnosis of depression in this study was based on subjective questionnaires rather than pathological or immunohistochemical examination. However, this practical clinical condition further reflects the importance of a precise, objective, and quantitative assessment in the diagnosis and evaluation of psychiatric disorders.

In conclusion, this study demonstrated that DLR can significantly improve the SNR of DTI images, significantly influencing the quantification of DTI-derived parameters. Compared to the ORI DTI, the fslmeants DLR DTI identified more depression-related WM damages and showed superior diagnostic performance in distinguishing mild-to-moderate from severe depression based on FA values of these detected damages. Therefore, the application of DLR to DTI may be beneficial for the assessment and management of patients with depression.

## Data Availability

The data that support the findings of this study are available from the corresponding author upon reasonable request.
