# Supplementary material for: Deep learning-based image reconstruction benefits diffusion tensor imaging for assessing severity of depression
Source: Front Neurosci. 2025 Aug 12;19:1607130. doi: 10.3389/fnins.2025.1607130 (PMC12378163; doi:10.3389/fnins.2025.1607130)
Supplement: Supplementary file 1 [file Data_Sheet_1.docx]

**Supplementary**

**
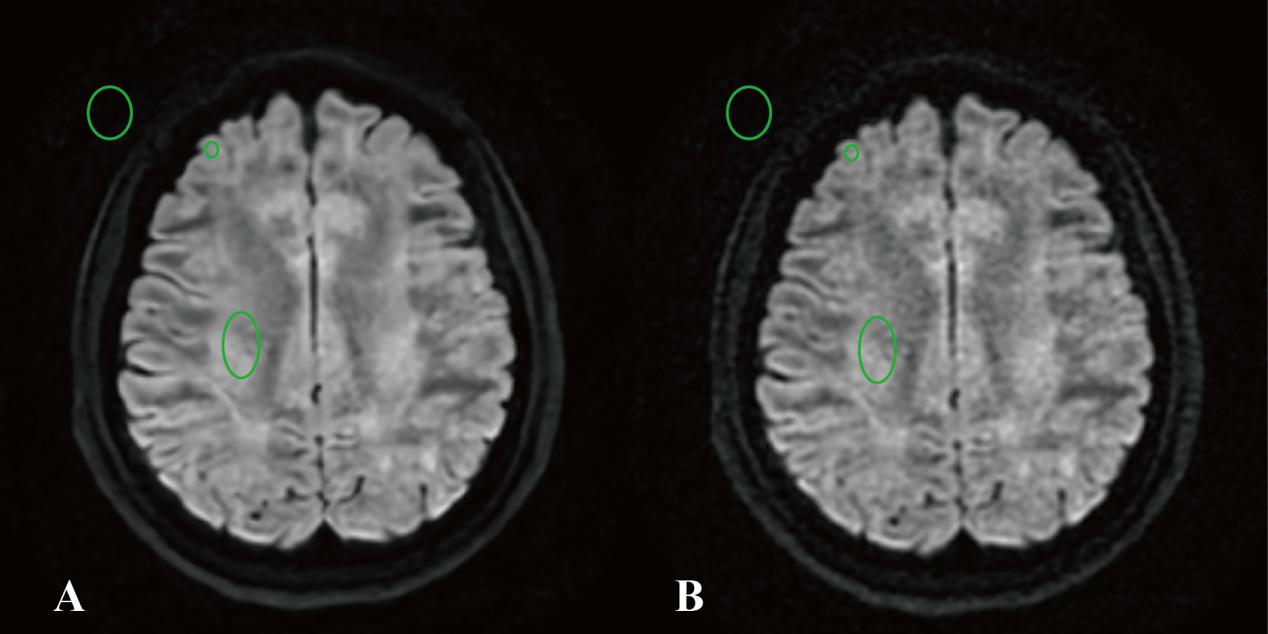
**

**Supplementary Figure 1. Illustration of region of interest (ROI) delineation for signal measurement.** A represents the original diffusion tensor imaging (DTI), and B represents the DTI reconstructed after deep learning post-reconstruction at the same slice. ROIs were delineated in the white matter of the right semi-oval center, the frontal cortex at the same slice, and the same location in the right frontal background of the image in both images. The green circles indicate the locations of the ROIs.

**Supplementary Table 1. The significantly different regions of white matter in skeletonized FA between the DLR and ORI DTI (DLR DTI < ORI DTI, Cluster voxel > 50)**

| **Cluster** | **Voxel size** | **MNI coordinate** | | | **P value** |
| --- | --- | --- | --- | --- | --- |
|  |  | **X(mm)** | **Y(mm)** | **Z(mm)** |  |
| 1 | 86785 | 91 | 81 | 19 | <0.001 |
| 2 | 319 | 61 | 68 | 47 | 0.002 |
| 3 | 261 | 120 | 79 | 43 | <0.001 |
| 4 | 260 | 71 | 52 | 67 | 0.002 |
| 5 | 86 | 100 | 77 | 23 | 0.004 |
| 6 | 57 | 56 | 61 | 26 | 0.011 |

ORI DTI: original diffusion tensor imaging; DLR DTI: deep learning-based image reconstruction diffusion tensor imaging; MNI: Montreal Neurological Institute.

**Supplementary Table 2. The significant differences in the extract values of FA in the different regions between DLR and ORI DTI (DLR DTI < ORI DTI, Cluster voxel > 50)**

| **Clusters** | **DLR DTI** | **ORI DTI** | **P value** |
| --- | --- | --- | --- |
| 1 | 0.488±0.014 | 0.500±0.014 | **<**0.001 |
| 2 | 0.249±0.019 | 0.259±0.020 | **<**0.001 |
| 3 | 0.243±0.017 | 0.254±0.018 | **<**0.001 |
| 4 | 0.359±0.031 | 0.376±0.031 | **<**0.001 |
| 5 | 0.251±0.029 | 0.267±0.031 | **<**0.001 |
| 6 | 0.239±0.022 | 0.249±0.027 | **<**0.001 |

ORI DTI: original diffusion tensor imaging; DLR DTI: deep learning-based image reconstruction diffusion tensor imaging.

**Supplementary Table 3. The performance of the models validated by leave-one-out cross-validation**

| **Model** | **Area under the curve** | **Accuracy** | **Sensitivity** | **Specificity** |
| --- | --- | --- | --- | --- |
| ORI_Right corticospinal tract model | 0.719 | 0.673 | 0.750 | 0.583 |
| DLR_Right corticospinal tract_1 model | 0.810 | 0.692 | .0714 | 0.667 |
| DLR_Right anterior thalamic radiation model | 0.881 | 0.808 | 0.857 | 0.75 |
| DLR_Left superior longitudinal fasciculus model | 0.807 | 0.731 | 0.750 | 0.708 |
| DLR_Right corticospinal tract_2 model | 0.795 | 0.731 | 0.786 | 0.667 |
| DLR_Combined model | 0.885 | 0.808 | 0.792 | 0.821 |

ORI: original; DLR: deep learning-based image reconstruction; corticospinal tract_1: corticospinal tract cluster 1; corticospinal tract_2: corticospinal tract cluster 2.


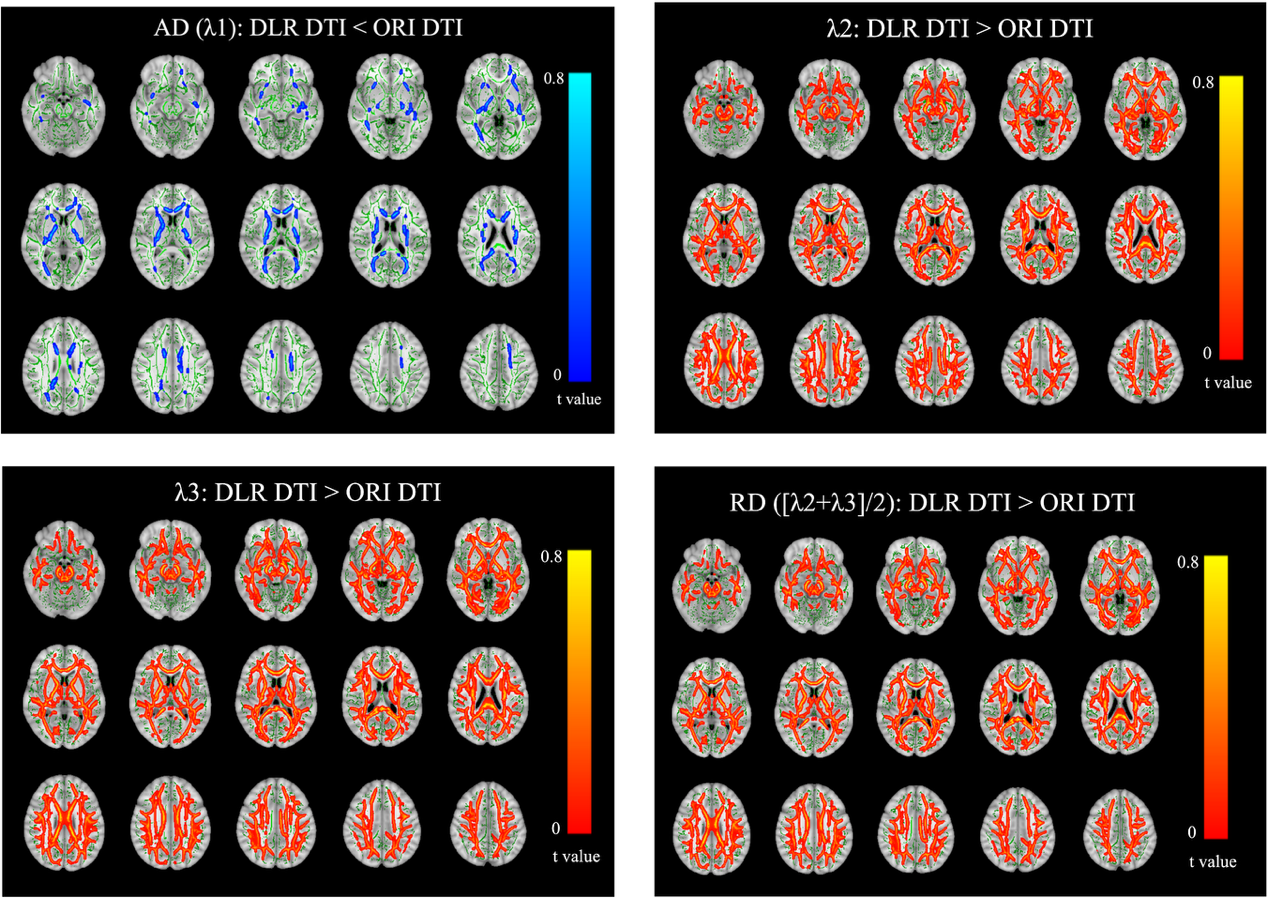


**Supplementary Figure 2. The comparation of AD, RD, λ2, and λ3 between DLR DIT and ORI DTI.** Deep learning-based image reconstruction diffusion tensor imaging (DLR DTI) showed a higher λ2 and λ3 in most white matter tracts, which results a higher radial diffusivity (RD). However, it was demonstrated that DLR DTI has lower axial diffusivity (AD). AD: axial diffusivity; ORI DTI: original diffusion tensor imaging; DLR DTI: deep learning-based image reconstruction diffusion tensor imaging; RD: radial diffusivity.
